# Effects of postpartum acetylsalicylic acid on uterine diseases and reproductive performance in dairy cattle

**DOI:** 10.3168/jdsc.2020-0047

**Published:** 2021-01-22

**Authors:** A.A. Barragan, S. Bas, E. Hovingh, L. Byler

**Affiliations:** 1 Department of Veterinary and Biomedical Sciences, Pennsylvania State University, University Park 18602; 2 Phytobiotics Futterzusatzstoffe GmbH Bvd, Villa Maria, Córdoba 5220, Argentina

## Abstract

Uterine diseases are some of the most prevalent and costly diseases for the dairy industry. It has been reported that an exacerbated and prolonged inflammatory response around calving can increase the risk of developing metritis, which subsequently can impair production and fertility in dairy cows. The objective of this study was to assess the effects of 2 oral administrations of acetylsalicylic acid 24 h apart on the incidence of uterine diseases [i.e., retained fetal membranes, clinical metritis, clinical endometritis) and reproductive performance [i.e., days in milk to conception, number of services to conception, proportion of cows pregnant at first service, proportion of abortion at first service (ABRT)] in dairy cows after calving. Cows from one dairy farm (n = 246) located in central Pennsylvania were included in this study. Animals were blocked by parity and randomly assigned to 1 of 2 groups: (1) ASA (n = 121): within ~12 h after parturition, cows received 2 treatments with acetylsalicylic acid (ASA; 200 mg/kg, 4 boluses) 24 h apart; or (2) UNT (n = 125): cows remained untreated. Clinical metritis was assessed at 7 ± 3 and 14 ± 3 d in milk (DIM) using a Metricheck device (Simcro Tech Ltd., Hamilton, New Zealand). Cows that presented a fetid, red-brownish, watery vaginal discharge were classified as having clinical metritis. Clinical endometritis was assessed through ultrasonography and defined as presence of hyperechoic fluid (>3 mm) in the uterus lumen at 50 ± 10 DIM. The incidence of retained fetal membranes was defined as the failure of expulsion of the placenta within 24 h after calving and recorded by farm personnel. The data were analyzed using the MIXED and GLIMMIX procedures of SAS (SAS Institute Inc., Cary, NC) as a randomized complete block design. A lower proportion of cows treated with acetylsalicylic acid developed clinical metritis at 7 ± 3 DIM (ASA = 34.97 ± 5.57%; UNT = 57.21 ± 5.80%) and tended to develop clinical endometritis at 50 ± 10 DIM (ASA = 3.84 ± 2.67%; UNT = 13.61 ± 5.23%) compared with cows that remained untreated. Cows treated with acetylsalicylic acid tended to require 18 d less to conceive compared with UNT cows (ASA = 125.63 ± 8.21 d; UNT = 143.44 ± 6.28 d). Furthermore, there was a tendency for a treatment × ABRT interaction, whereby ASA cows that aborted at first service tended to conceive 35 d sooner compared with UNT cows that aborted at first service (ASA = 151.42 ± 15.90 d; UNT = 186.38 ± 11.84 d). Although there was no difference in pregnancy per AI at first service between treatment groups (ASA = 23.88 ± 7.92%; UNT = 17.93 ± 6.31%), the proportion of cows aborting at first service in the ASA group tended to be lower than that in the UNT group (ASA = 1.03 ± 1.06%; UNT = 6.04 ± 2.62%). Results from this study suggest that a short-duration acetylsalicylic acid treatment after calving may decrease the incidence of uterine diseases and improve reproductive success in cows that aborted at first service in dairy cattle.

## Highlights

• Treated cows had lower incidence of uterine diseases
• Treated cows tended to have a lower abortion rate at first service
• Treated cows that aborted at first service tended to conceive 35 days sooner

## Graphical Abstract

**Figure:**
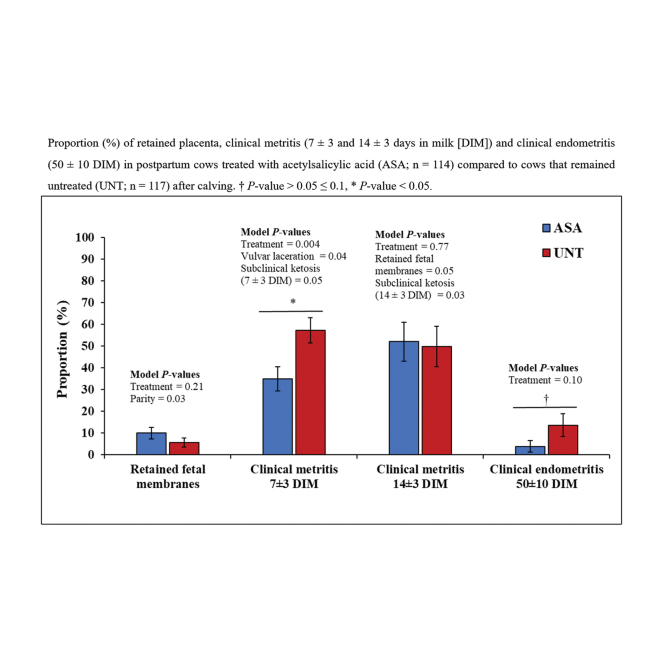
**Summary:** The postpartum period is a challenging time for dairy cows, as they experience physiological challenges, such as systemic and metabolic inflammation, which increase the risk of diseases and poor subsequent production and fertility. The main objective was to assess the effects of oral administration of acetylsalicylic acid after calving on uterine diseases and reproductive performance of postpartum dairy cows. Cows treated with acetylsalicylic acid had lower incidence of clinical metritis and endometritis and improved reproductive performance. Results from this study suggest that a treatment with acetylsalicylic acid after calving may improve health and fertility in dairy cattle.

The peri-parturient period is one of the most challenging times for dairy cows because they must cope with physiological challenges such as decreased dry matter intake, impaired immune system functions, and increased metabolic and systemic inflammation (Drackley, 1999; LeBlanc, 2010). There is some evidence that suggests that an exacerbated stressful or inflammatory response around calving may increase the risk of metabolic and infectious diseases. For instance, Goff and Horst (1997) reported that cows that experienced subclinical or clinical hypocalcemia 48 h after calving had a higher concentration of cortisol at calving when comparing with cows that were normocalcemic. Other authors found that cows that developed either a mild or severe case of clinical metritis within 21 d after calving had higher concentrations of inflammatory biomarkers (i.e., haptoglobin, **HP**) at calving (Huzzey et al., 2009). Similarly, Barragan et al. (2020b) reported that cows that had one or more clinical disease events in the first 60 DIM experienced higher inflammation and stress in the first week after calving compared with cows that remained healthy. There is also scientific evidence that suggests that high inflammation in early lactation can directly affect cow fertility. Cheong et al. (2017) observed that regardless of uterine bacterial contamination, resumption of ovarian cyclicity started earlier in cows with lower circulating HP concentrations in the first 3 DIM.

Clinical metritis (**CM**), defined as the presence of an enlarged uterus and an abnormal red-brownish fetid-smelling vaginal discharge with or without system clinical signs such as fever within 21 d after calving (Barragan et al., 2018), is one of the most common diseases in dairy cows, affecting between 15 and 20% of post-parturient animals (Gilbert, 2016). Clinical metritis negatively affects welfare and production of cows due to increased systemic inflammation and pain (Barragan et al., 2018) and decreased milk yield (Rajala and Gröhn, 1998). However, one of the most detrimental effects of uterine diseases in cow performance is the decrease in fertility experienced by affected animals. Fourichon et al. (2000) performed a meta-analysis of the effects of CM on reproduction and reported that overall cows that experienced CM received their first service 7 d later and had a 20% lower conception rate compared with healthy animals. Nevertheless, having uterine disease or non-uterine disease (e.g., mastitis) early in lactation may affect fertility in the same degree. Ribeiro et al. (2016) reported that cows that experienced either uterine diseases or non-uterine diseases had decreased pregnancy per breeding and increased pregnancy losses.

The use of nonsteroidal anti-inflammatory drugs (**NSAID**) after calving aimed at improving welfare and performance of dairy cows has been widely investigated in recent years. Although several authors reported fairly similar results regarding milk yield in cows treated with these drugs after calving (Farney et al., 2013b; Carpenter et al., 2016; Barragan et al., 2020a), their effects on disease prevention and reproduction are more inconsistent. For instance, Newby et al. (2017) reported that cows treated immediately after calving and 24 h later with flunixin meglumine had higher incidence of retained fetal membranes (**RFM**) and metritis. Nonsteroidal anti-inflammatory drugs work by inhibiting prostaglandins production, which plays an important role in the series of events involved in parturition and expulsion of fetal membranes (Laven and Peters, 1996). Therefore, administration during calving, or immediately after, with powerful NSAIDs may have the potential to greatly reduce prostaglandin synthesis, which could lead to RFM and increased risk of other uterine diseases such as CM. Although flunixin meglumine is the only Food and Drug Administration (**FDA**)-approved NSAID for use in lactating cattle in the United States (Coetzee, 2013), there is an ethical responsibility to provide proper care alternatives for animals in discomfort. Other authors assessed the effects of a weak NSAID (i.e., acetylsalicylic acid) on cow health, without finding difference in disease incidence between treated and control animals (Barragan et al., 2020a,c). Because acetylsalicylic acid and its derivatives are not approved by the FDA for use in lactating dairy cattle in the United States, dairy producers must consult with a licensed veterinarian before implementing treatments utilizing these products.

On the other hand, studies that assessed the effects of postpartum NSAID administration on cow reproductive performance reported no effects (Meier et al., 2014), negative effects (Farney et al., 2013b), or positive effects (Bertoni et al., 2004; Barragan et al., 2020a). For instance, Meier et al. (2014) found no effect of early (1–5 d after calving) or late (19–23 d after calving) postpartum anti-inflammatory treatment (i.e., carprofen) on conception or pregnancy rates. Other authors (Bertoni et al., 2004), who treated cows with injectable lysine acetylsalicylate for 5 d after calving, reported that treated cows had improved rates of pregnancy at first service. Similarly, Barragan et al. (2020a) reported that cows treated with 4 doses of oral boluses of acetylsalicylic acid every 12 h after calving tended to require fewer days and required fewer services to conceive compared with placebo cows. Therefore, to elucidate the actual effects of anti-inflammatory treatment after calving on disease prevention and cow fertility, more research is warranted.

The objective of this study was to assess the effects of oral administration of 2 doses of acetylsalicylic acid 24 h apart on the incidence of uterine diseases in the first 60 d after calving [i.e., RFM, CM, and clinical endometritis (**CE**)] and reproductive performance [i.e., days in milk to conception (**DIMC**), number of services required to conceive (**SPC**), proportion of cows pregnant at first service (**PFS**), and abortion at first service (**ABRT**)] in dairy cows. We hypothesized that cows treated with acetylsalicylic acid would have lower incidence of uterine diseases, and subsequently, better reproductive performance than untreated cows.

Postpartum dairy cows (n = 246) from a 700-cow dairy farm located in central Pennsylvania were enrolled in this study. The treatment administration and field data collection components of this study were performed from May 2018 to September 2018, and experimental units were followed until their lactation was completed or they died or were sold. Farm facilities and management practices are described elsewhere (Barragan et al., 2020c). The voluntary waiting period was 70 ± 3 DIM. The farm reproductive management consisted of a combination of a Presynch [i.e., 2 prostaglandin (**PG**) doses separated by 14 d starting at 42 DIM], with estrus detection and AI after the second PG dose, and Ovsynch protocols [i.e., a GnRH dose followed by 2 PG doses (24 h apart) 7 d later, then a second GnRH dose 56 h later followed by timed AI 16 h after] for first service. Pregnancy diagnosis was performed weekly by the farm veterinarian through transrectal ultrasonography at 31 ± 3 and 55 ± 3 d after breeding. The ovarian structures of cows diagnosed as nonpregnant were assessed at pregnancy diagnosis, and the resynchronization protocol for these animals was selected based on the presence or absence of a corpus luteum (**CL**). Briefly, cows that had a CL received a PG dose and were AI if they showed estrus within 14 d; if cows did not show estrus after 14 d, they were enrolled in an Ovsynch protocol. Cows without CL were enrolled in an Ovsynch protocol at pregnancy diagnosis. All breeding was performed by the farm herd manager, and cows that did not conceive after 6 services were classified as “do not breed” and were no longer eligible for insemination.

Within ~12 h after parturition, the study team blocked cows by parity [i.e., primiparous = 77 (ASA = 39; UNT = 38); multiparous = 169 (ASA = 82; UNT = 87)] and assigned them randomly (simple randomization method, flipping the coin; Kang et al., 2008) to 2 groups: (1) **ASA** (n = 121): cows received 2 treatments with acetylsalicylic acid (4 boluses of 480 grain, estimated average dose per BW = 200 mg/kg; Agri Laboratories, St. Joseph, MO) 24 h apart; or (2) were untreated (**UNT**; n = 125). Oral bolus treatments were administered, using headlocks to restrain cows, by the farm herd manager who was previously trained by A. A. Barragan. The first oral administration was provided at enrollment, and the second administration was provided 24 h later. Treatments were performed twice daily, in the morning (starting at 0900–1000 h) and in the afternoon (starting at 1700–1800 h). Cows in the UNT group were restrained at the same times as the ASA cows for blood sample collection. Retention of fetal membranes was defined as the failure of expulsion of the placenta within 24 h after calving and was assessed by farm personnel. Data regarding RFM incidence was collected from the on-farm computer records (PCDART, North Carolina State University, Raleigh, NC). Assessment of CM was performed by the research team, who was blinded to treatment, at 7 ± 3 and 14 ± 3 DIM using a Metricheck device (Simcro Tech Ltd., Hamilton, New Zealand), based on the color, proportion of pus, and odor of the vaginal discharge, as follows: (1) clear fluid, (2) <50% white purulent fluid, (3) ≥50% white purulent fluid, (4) red-brownish watery without fetid smell fluid, and (5) fetid, red-brownish, watery fluid (Barragan et al., 2019). Cows with a fetid, red-brownish, watery vaginal discharge (score 5) were classified as having CM. Transrectal ultrasonography was performed by the research team, who was blinded to treatment, using an ultrasound with a linear prove (Evo, Ibex, E.I. Medical Imaging, Loveland, CO) at 50 ± 10 DIM for assessment of CE [defined as the presence (>3 mm diameter) of abnormal fluid (i.e., hyperechoic fluid); Barlund et al., 2008]. Body condition score and subclinical ketosis assessment was described elsewhere (Barragan et al., 2020a). Measures of reproductive performance parameters (i.e., DIMC, SPC, PFS, ABRT), as well as data regarding incidence of clinical diseases in the first 60 DIM diagnosed by farm personnel (a complete disease list, including disease definition and incidence, can be found in Barragan et al., 2020c), were obtained from the on-farm computer records. The farm personnel involved in the clinical disease diagnoses and reproductive management of study cows were blinded to treatment. The experimental procedures described above were approved by the Institutional Animal Care Use Committee at The Pennsylvania State University (Protocol number 201800280).

Statistical analyses of this complete randomized blocked design study were performed using SAS software (version 9.4, SAS Institute Inc., Cary, NC). A sample size calculation was provided elsewhere (Barragan et al., 2020c). Cow was used as the experimental unit. The homogeneity of variances and normality of the dependent continuous variables were assessed using graphical methods (histogram and Q-Q plot), Bartlett's test, and Shapiro-Wilk statistic using the UNIVARIATE procedure of SAS.

Fifteen cows left the herd before 60 DIM (ASA died = 4, UNT died = 5; ASA sold = 3, UNT sold = 3) and were excluded from the experiment. Therefore, a total of 114 (multiparous = 76; primiparous = 38) and 117 (multiparous = 81; primiparous = 36) cows remained in the ASA and UNT treatments, respectively. There was no difference in the proportion of cows that experienced retained placenta (*P* = 0.21; ASA = 9.97 ± 2.75 %; UNT = 5.59 ± 2.06 %) or CM at 14 ± 3 DIM (*P* = 0.77; ASA = 52.10 ± 8.95 %; UNT = 49.81 ± 9.35 %) between treatment groups (Figure 1). A lower proportion of cows treated with ASA developed CM at 7 ± 3 DIM (*P* = 0.004; ASA = 34.97 ± 5.57 %; UNT = 57.21 ± 5.80 %) and tended to develop CE at 50 ± 10 DIM (*P* = 0.10; ASA = 3.84 ± 2.67 %; UNT = 13.61 ± 5.23 %) compared with cows that remained untreated (Figure 1).

**Figure 1:**
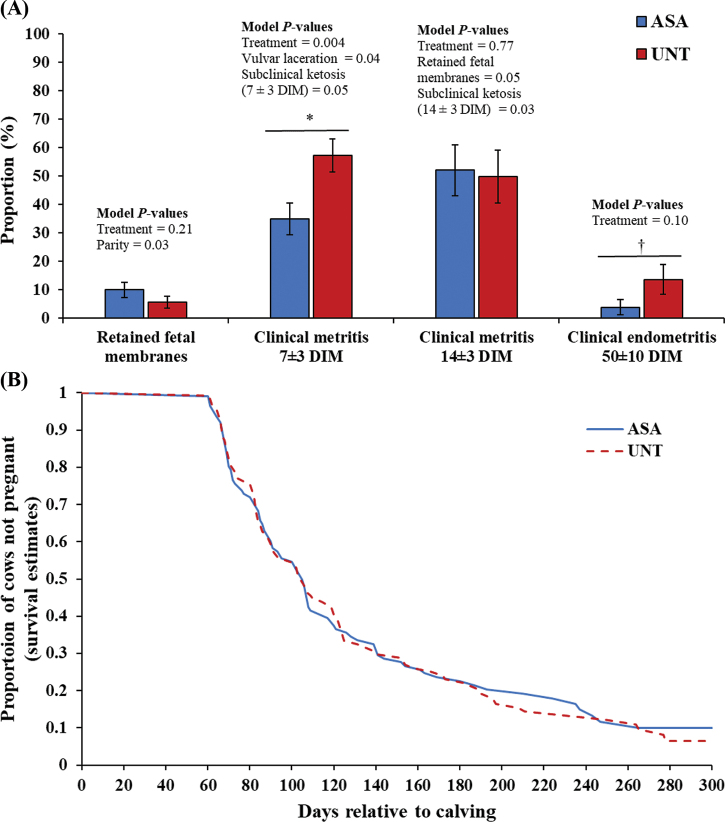
(A) Proportion (%) of retained placenta, clinical metritis (7 ± 3 and 14 ± 3 DIM), and clinical endometritis (50 ± 10 DIM) in postpartum cows treated with acetylsalicylic acid (ASA; n = 114) compared with cows that remained untreated (UNT; n = 117) after calving. *P < 0.05, †0.05 < *P* ≤ 0.1. Error bars indicate SEM. (B) Survival curve for time to conception after the voluntary waiting period of dairy cows treated with ASA (solid blue line; n = 114) compared with UNT cows (red dotted line; n = 117). The adjusted hazard ratio (AHR 95% CI) for pregnancy (*P* = 0.83) was 0.97 for ASA cows (referent = UNT cows). Mean time to pregnancy was 130.4 d (131–235 d) and 130.5 d (131–196 d) for ASA and UNT cows, respectively.

Two statistical analyses were performed for DIMC (Barragan et al., 2020a). First, DIMC was analyzed using multivariable mixed linear regression through the MIXED procedure of SAS. The variables originally offered to the model were treatment, parity, single clinical disease events diagnosed by farm personnel (e.g., dystocia; complete list available in Barragan et al., 2020c), BCS (at enrollment, 7 ± 3 DIM, 14 ± 3 DIM, 50 ± 10 DIM), subclinical ketosis (7 ± 3 DIM, 14 ± 3 DIM), CM (7 ± 3 DIM, 14 ± 3 DIM), CE, and ABRT, as well as 2-way interactions between significant main variables. The Wald backward selection criterion (*P* > 0.15) was used to remove nonsignificant variables from the model. Treatment variable was forced in the models, and cow was included in the model as random effect in the RANDOM statement. The variables that remained in this model were treatment, parity, CM (14 ± 3 DIM), ABRT, and treatment × ABRT interaction. The results are presented as least squares means (LSM) and standard error of the mean (SEM), calculated and adjusted with Tukey-Kramer method using the “lsmeans” statement. When the interaction between treatment and a variable was significant (*P* ≤ 0.1), the “slice” option in the “lsmeans” statement was used to determine differences among treatments on each level (single comparison) of the interacting variable. Second, a Cox proportional hazard model was used to assess DIMC up to 300 DIM in a survival analysis using the PHREG procedure of SAS. The variables offered to this model were the same as for the mixed model. The variables that remained were treatment, parity, RFM, CM (14 ± 3 DIM), and ABRT. The LIFETEST procedure of SAS was used to obtained data output to develop a survival curve graphic in Excel (Microsoft Corp., Redmond, WA) to depict the proportion of cows pregnant up to 300 DIM (Figure 2).

Due to failure to achieve normal distribution after data transformation (log_10_, R2, root square transformation), SPC was analyzed using the nonparametric Kruskal-Wallis test through the NPAR1WAY procedure of SAS. Median and interquartile range values are reported.

The proportion of cows that experienced RFM, CM, and CE, as well as PFS and ABRT, were analyzed by multivariable logistic regression using the GLIMMIX procedure of SAS. The Wald statistic backward selection criterion (*P* > 0.15) was used to identify significant variables that remained in the model. Treatment variable was forced in the models, cow was included as random effect, and the same variables as for the mixed model were originally offered. For RFM, the variables that remained in the model were treatment and parity. For CM at 7 ± 3 DIM, treatment, vulvar laceration (single clinical disease from farm records) and subclinical ketosis (7 ± 3 DIM) remained in the model; whereas for CM at 14 ± 3 DIM, treatment, RFM (single clinical disease from farm records) and subclinical ketosis (14 ± 3 DIM) remained in the model. For CE, only treatment variable remained in the model. For the PFS and ABRT, the variables that remained in the model were treatment and CM (14 ± 3 DIM). The variables of interest as well as their interactions were considered significant if *P* < 0.05; *P* > 0.05 ≤ 0.10 was considered a tendency.

Cows treated with ASA tended to require 18 fewer days to conceive compared with UNT cows (*P* = 0.08; ASA = 125.63 ± 8.21 d; UNT = 143.44 ± 6.28 d; Table 1). However, the Cox proportional hazard analysis showed no difference in the hazard to conception in the first 300 DIM between treatment groups (*P* = 0.25; Table 2; Figure 2). Furthermore, there was a tendency (*P* = 0.09) for a treatment × ABRT interaction, where ASA cows that aborted at first service tended to conceive 35 d sooner compared with UNT cows that aborted at first service (ASA = 151.42 ± 15.90 d; UNT = 186.38 ± 11.84 d; Table 1). Although there was no difference in PFS between treatment groups (*P* = 0.40; ASA = 23.88 ± 7.92 %; UNT = 17.93 ± 6.31 %; Table 1), the proportion of cows aborting at first service (ABRT) tended to be lower in the ASA group than in the UNT group (*P* = 0.10; ASA = 1.03 ± 1.06 %; UNT = 6.04 ± 2.62 %; Table 1). The SPC was not different between study groups (*P* = 0.23; ASA = median of 2 services; interquartile range of 1–3 services; UNT = median of 2 services; interquartile range of 1–3 services).

**Table 1:** Time (DIM) to conception for all cows (LSM ± SEM), DIM to conception by abortion at first service (LSM ± SEM), pregnancy per AI at first service (%; LSM ± SEM) and pregnancy loss at first service (%; LSM ± SEM) in postpartum cows treated with acetylsalicylic acid (ASA; n = 114) compared with untreated cows (UNT; n = 117) after calving

Variable	Level	Treatment		*P*-value
		ASA	UNT	
DIM to conception		125.63 ± 8.21	143.44 ± 6.28	0.08
DIM to conception by abortion at first service	Not aborted	99.84 ± 4.03	100.50 ± 3.89	0.90
	Aborted	151.42 ± 15.9	186.38 ± 11.84	0.07
Pregnancy per AI at first service		23.88 ± 7.92	17.93 ± 6.31	0.40
Pregnancy loss at first service		1.03 ± 1.06	6.04 ± 2.62	0.10

**Table 2:** Final Cox proportional hazard model for DIM to conception in postpartum cows treated with acetylsalicylic acid (ASA; n = 114) compared with cows that remained untreated (UNT; n = 117) after calving

Variable	Level	Coefficient	SE	Hazard ratio	*P*-value
Treatment	ASA	0.18	0.16	1.20	0.25
Parity	Multiparous	−0.59	0.17	0.55	0.0007
Retained fetal membranes	Yes	−0.57	0.31	0.56	0.06
Metritis at 14 DIM	Yes	−1.13	0.18	0.32	<0.0001
Abortion at first service	Yes	−1.45	0.30	0.23	<0.0001

The main findings of this study were as follows: (1) cows treated with ASA had lower incidence of CM at 7 ± 3 DIM and CE at 50 ± 10 DIM compared with UNT cows; and (2) ASA cows tended to require fewer days to conceive and have lower abortions at first service compared with UNT cows.

In postpartum cows, CM is a prevalent condition that not only affects health and performance of cows but also welfare (Fourichon et al., 2000b; Gilbert, 2016; Barragan et al., 2018). It has been reported that cows that experience higher inflammation around calving, assessed by high concentration of HP, may be at a higher risk of developing infectious diseases such as CM (Huzzey et al., 2009). McCarthy et al. (2016) assessed the degree of inflammatory response in cows during the first week of lactation and grouped them into inflammatory quartiles (i.e., Q1, Q2, Q3, Q4), reporting that cows in Q1 had improved neutrophil and monocyte functions (i.e., oxidative burst). Therefore, treating cows after calving to modulate the systemic inflammatory response could be an effective approach to improve immune system functions and aid in preventing CM in dairy cattle. Pascottini et al. (2020) reported that treatment with an NSAID (i.e., meloxicam) decreased HP concentrations from d 2 until d 4 of treatment (i.e., 11–13 DIM) and improved neutrophil function of postpartum cows. Nevertheless, in the present study there were no difference in HP concentrations between treatment groups at 30 ± 6 h and 7 ± 3 d in the study animals (reported elsewhere; Barragan et al., 2020c). Because of substantial and rapid physiological changes in HP concentration around calving (Uchida et al., 1993; Barragan et al., 2020b), the large range in sampling times could explain, in part, the lack of differences in HP concentrations. Other authors (Barragan et al., 2020b) reported that multiparous cows that were treated within 12 h after calving with 4 oral administrations of acetylsalicylic acid 12 h apart had lower HP concentrations compared with multiparous cows treated with a placebo. Similarly, Pascottini et al. (2020), which sampled postpartum cows more frequently (once a day) and used another NSAID (i.e., meloxicam), reported that treated cows had decreased HP concentrations compared with control cows. Nevertheless, neither Barragan et al. (2020a) nor Pascottini et al. (2020) reported a difference in uterine disease incidence. These differences could be explained at least partly by the different treatment strategy and disease diagnosis methods used in the latter studies. For instance, Barragan et al. (2020a) used farm records to assess uterine disease incidence, which can often reflect underdiagnosis of mild cases of disease. In contrast, Pascottini et al. (2020) treated cows starting at 10 DIM, when the calving-related inflammatory response that may affect more severely uterine health (Huzzey et al., 2009) starts to decrease (Uchida et al., 1993; Barragan et al., 2020b).

The effects of NSAIDs on cow fertility has been previously reported; however, there is inconsistency in findings. For instance, Bertoni et al. (2004) reported that cows treated with injectable lysine acetylsalicylate for 5 d after calving required fewer services to become pregnant and fewer cows became repeat breeders compared with control animals. More recently, Barragan et al. (2020a) reported that postpartum cows (within 12 h after calving) treated with 4 oral administrations of acetylsalicylic acid 12 h apart tended to require 12 d less to conceive and fewer services to conceive compare with placebo cows. Although the latter results agree in part with the findings observed in the present study, the Cox proportional hazard analysis did not show differences between treatment groups in the current study. This could imply that there may be a weaker association between treatment and DIMC in this cohort than in others (Barragan et al., 2020a). In addition, the current study reported improvements in fertility in ASA-treated cows that aborted at first service, which has not been reported before. Nevertheless, other authors failed to show differences (Carpenter et al., 2016) in fertility in cows treated with NSAIDs or reported negative effects (i.e., longer return to estrus in first lactation cows; Farney et al., 2013b). Bertoni et al. (2008) reported that lower inflammation after calving reduced incidence of metritis and improved reproductive performance (i.e., fewer days open and tended to require fewer services to conceive). Supporting these findings, other authors (Cheong et al.,2017) observed that regardless of uterine bacterial contamination, cyclicity ovarian resumed earlier in cows with lower inflammation at 3 DIM. In addition, Farney et al. (2013a) observed that long (7 d) treatment with NSAID of postpartum cows increased cow metabolic stress (i.e., high concentration of BHB). The negative effects of uterine diseases and high concentration of BHB on cow fertility are well documented (Fourichon et al., 2000b; Walsh et al., 2007; LeBlanc, 2010; Ribeiro et al., 2013). In the present study, cows treated with ASA had a lower concertation of BHB at 14 ± 3 DIM and higher BCS by 50 ± 10 DIM compared with UNT cows (reported elsewhere; Barragan et al., 2020c). The ASA treatment strategy might have allowed for a more efficient energy expenditure, decreasing cow metabolic stress and improving cow fertility.

In conclusion, the results of the present study suggest that a short-duration treatment strategy with ASA may decrease the incidence of clinical uterine diseases and improve reproductive success in cows that aborted at first service in dairy cattle. However, readers must be aware of the regulatory conditions regarding the use of acetylsalicylic acid (not approved by the FDA for use in lactating cattle in the United States) and its derivatives in dairy cattle, including milk and meat withdrawal times (i.e., 24 h; Smith et al., 2008), and must consult with a licensed veterinarian before implementing any treatment utilizing these products. Further research should be aimed at replicating the positive effects of NSAID treatments after calving on cow fertility in larger populations and identifying specific biological processes that may be involved with these effects.
